# Stress in the Educational System as a Potential Source of Epigenetic Influences on Children's Development and Behavior

**DOI:** 10.3389/fnbeh.2018.00143

**Published:** 2018-07-13

**Authors:** Daniel Frías-Lasserre, Cristian A. Villagra, Carlos Guerrero-Bosagna

**Affiliations:** ^1^Instituto de Entomología, Universidad Metropolitana de Ciencias de la Educación, Santiago, Chile; ^2^Department of Physics, Chemistry and Biology (IFM), Linköping University, Linköping, Sweden

**Keywords:** epigenetics, stress, learning, cultural inheritance, education

## Abstract

Despite current advances on the relevance of environmental cues and epigenetic mechanisms in biological processes, including behavior, little attention has been paid to the potential link between epigenetic influences and educational sciences. For instance, could the learning environment and stress determine epigenetic marking, affecting students' behavior development? Could this have consequences on educational outcomes? So far, it has been shown that environmental stress influences neurological processes and behavior both in humans and rats. Through epigenetic mechanisms, offspring from stressed individuals develop altered behavior without any exposure to traumatizing experiences. Methylated DNA and noncoding RNAs regulate neurological processes such as synaptic plasticity and brain cortex development in children. The malfunctioning of these processes is associated with several neurological disorders, and these findings open up new avenues for the design of enriched environments for education and therapy. In this article, we discuss current cases of stress and behavioral disorders found in youngsters, and highlight the importance of considering epigenetic processes affecting the development of cognitive abilities and learning within the educational environment and for the development of teaching methodologies.

## Introduction

Anyone who has watched the movie “Whiplash” (2014) must certainly have been impressed by the passion and devotion that 19-year-old Andrew puts into excelling as a drummer. However, “Whiplash” is much more than a movie about a musician's devotion to playing his instrument. Rather it is a movie that contrasts two types of educational concepts: one that compassionately supports individuals toward learning new skills and focuses on providing emotional and financial support, and another whose concept of education is based on selecting the “best in the class” and demanding progress from them by placing them in high-stress situations. What is particular about “Whiplash” is that Andrew is simultaneously subjected to these two types of educational models. Interestingly, supporters of each teaching model could claim Andrew succeeded because of their tactics: he could have exceled due to the strict education program imposed by his music teacher Terence Fletcher, or due to the love, confidence and support provided by his ever-present father.

Although strict education certainly succeeds in creating some kind of “excellence,” it does so at a very high cost: many will drop out during the process (as happens in “Whiplash,” in which there is even a suicide), while the survivors that excel will do so with a legacy of trauma in their brains (as is the case with Andrew). On the other hand, how many dropouts could be avoided by applying the paradigm of compassionate education, combined with an enriching environment? Epigenetic mechanisms of regulation and gene expression changes have much to say about the effects of both trauma and stress on human biology, as well as about the influence of enriched environments both on intermediate, transient dynamics (Dudley et al., 2011; Xie et al., 2013; Pizzimenti and Lattal, 2015), as well as on the long-term development and functioning of brain and behavior (Murgatroyd and Spengler, 2011; Crews et al., 2012; Denhardt, 2017).

Epigenetics is a contemporaneous discipline derived from genetics that includes the environmental context as a relevant part of heredity. Currently, it is strongly influencing a variety of fields, including Medicine, Psychiatry and Psychology (Hollar, 2016b; Mulder et al., 2017). Taking these fields into consideration, here we propose that Epigenetics will have a profound impact on Educational Sciences through the links between Epigenetics and Neurosciences. This paper explores early environmental and epigenetic influences in the development of human cognitive abilities. Here, we review major breakthroughs linking the epigenetic influences of stress to behavior and cognition, with special attention paid to education. As a counterpoint, we discuss the reversibility of such deleterious epigenetic effects, as has currently been demonstrated by the study of epigenetic marking in post-traumatic disorder (PSD) patients after therapy. We make the case of the potential benefits of an enriched, non-stressful educational environment on the development of cognitive capabilities during early stages of human ontogeny. We propose an interdisciplinary revision of educational methods that consider epigenetics. The aim of this is not only to avoid -stress-derived imprints, but also to propitiate the development of tools for healing and modifying potential detrimental epigenetic consequences emerging in the nervous system of children exposed to environmental stressors. The latter includes mistreatment, poverty and drug abuse, as well as stress susceptibility inherited from parental DNA epigenetic modifications. Overall, we highlight the need for consideration of how epigenetic inheritance mechanisms may influence human neurobiology in formal education. We describe the available evidence supporting the claim that strong selective regimes based on punishment and reward are detrimental for children and young people, and that those factors may negatively contribute to the development of learning capabilities and cognition through epigenetic mechanisms. Additionally, we show that epigenetic marks have the potential to be inherited, highlighting the impact of early development environmental inputs, especially for “educational environments.” Following that, we suggest intervening in formal education as a prospective window of ontogenetic experiences, in order to contribute with healthy epigenetic dynamics in the development of the human brain and behavior. Finally, as young *Homo sapiens*, from all cultures and credos, tend to experience rather standardized educational experiences, we consider that the link between epigenetic inheritance and human learning must be taken into serious consideration both in formal education, and as an academic venue for research related to educational sciences, neurobiology, genetics and human evolution.

## Epigenetic mechanisms of inheritance

Current scientific advancements have demonstrated the existence of certain chemical modifications in the DNA structure capable of regulating gene expression and of being mitotically stable. These chemical changes, currently known as “epigenetic modifications,” are found in many lineages in the Tree of Life, including our own species, and include at least five different known processes: (i) DNA methylation, hydroxymethylation and demethylation, (ii) rearrangements of the chromatin structure, histone modifications (e.g., acetylation, methylation, ubiquitination), (iii) RNA silencing mediated by non-coding RNAs (ncRNAs), RNA editing, epi and paramutations (Morgan et al., 2005; Probst et al., 2009; Gonzalez et al., 2011; Frías-Lasserre, 2012; Frías-Lasserre and Villagra, 2017), (iv) Mobile DNAs, such as transposable elements and endogenous viral particles (Kazazian, 2004; Cordaux and Batzer, 2009), and finally (v) a wide array of transcriptional agents, signaling molecules and gene regulatory factors, or environmental triggers (e.g., exposure to a given temperature or light cycle) (Zhang and Ho, 2011; Füllgrabe et al., 2013; Azzi et al., 2014; Weyrich et al., 2016). For instance, it has been demonstrated that novel stressors may act as environmental influences on organisms, driving the induction of epigenetic changes. It has been proposed that this begins with prompt, short-lived changes that may ultimately lead to stable epigenetic marks, in a multistep fashion. For instance, in mice it has been experimentally established that early life stress (ELS) by maternal separation induced histone acetylations capable of modifying the expression of the genes Arc and Egr1 in their hippocampus (involved in synaptic plasticity). These genes are related to the development of limbic brain circuits. These fast epigenetic modifications are paralleled by raises in dendritic complexity and spine number of hippocampal CA3 pyramidal neurons in animals suffering ELS in early ontogeny. Therefore, ELS induces activation of synaptic plasticity genes, mediated by epigenetic mechanisms. These events represent early steps in the adaption of neuronal networks to stressful environments, as they program the developing organism and influence the development of brain and behavior (Xie et al., 2013).

All these environmentally sensitive changes have been demonstrated to be of paramount relevance in the evolution of multicellular organisms including our own species, and are repeated in each ontogeny (Gottlieb, 1992; Oyama et al., 2003). Thus, these may define species' canonical traits by epigenetic processes (Reik and Walter, 2001; Salmon et al., 2008; Jablonka and Raz, 2009; Johannes et al., 2009; Koerner et al., 2009; Verhoeven et al., 2010; Daxinger and Whitelaw, 2012; MacDonald, 2012; Frésard et al., 2013). Alternatively, as it is the case of transient epigenetic modifications, these traits may lead to novel phenotypes or traits in the course of evolution (Gottlieb, 1992; Xie et al., 2013). Therefore, the change or maintenance of phenotypes through ontogeny it is depending on the coaction of different levels of organization within the organism (Gottlieb, 1992) from genetic and epigenetic activity to behavior, with the influence of the surrounding environment.

Regarding to the role of epigenetic modifications as part of the regular inheritance and function of organisms, several marvelous examples have been found. For instance, it has been discovered that ncRNAs, small ribonucleic acid molecules not bearing a proteosyntetic function (Ghildiyal and Zamore, 2009; Jacquier, 2009; Pauli et al., 2011), are capable of regulating gene expression at the transcriptional and post-transcriptional level (Wang et al., 2008), and are dynamic drivers of phenotypic change and biological diversity (Frías-Lasserre and Villagra, 2017). Strikingly, recent evidence shows that an altered ncRNA function gives rise to diverse hereditary maladies, including neurological disorders (Vučićević et al., 2014). In mice's early-life maltreatment due to abusive behavior by stressed caretakers that induces lasting DNA methylation changes in the brain-derived neurotrophic factor (BDNF) gene prompting its altered gene expression in the adult prefrontal cortex (Roth et al., 2009a).

Concerning the role of epigenetics in behavioral development, studies on mammal model organisms, such as rats, have demonstrated the existence of sensitive developmental periods of major epigenetic reprograming during ontogeny (Roth et al., 2009a; Hackett and Surani, 2013a). For instance, during prenatal and early neonatal folliculogenesis, major epigenetic modifications take place, involving whole genome remethylation (Walker and Ho, 2012). These steps are crucial in the formation of every tissue, in particular neural tissues, and have key roles in the conformation of behavior (Reik et al., 2001).

Two well described developmental intervals exist in which major epigenetic rearrangements occur in a mammal's genome (Hackett and Surani, 2013b). One of these periods occurs after fertilization, when a major reduction in DNA methylation occurs, followed by the re-establishment of DNA methylation patterns by the time of blastocyst implantation (Hackett and Surani, 2013a). This epigenetic reprogramming is pivotal for somatic cell line differentiation. Another high relevance phase of epigenetic reprogramming occurs during the migration of primordial germ cells (PGCs) toward their final establishment in the gonads (Allegrucci et al., 2005; Lees-Murdock and Walsh, 2008). During this phase, major demethylation of the genome also occurs, followed by re-methylation (Lees-Murdock and Walsh, 2008; Hackett and Surani, 2013a). It is important to highlight that these periods of resetting DNA methylation patterns, are windows of sensitivity to environmental exposures, both in PGCs and early embryos (Jirtle and Skinner, 2007; Feil and Fraga, 2012). However, affecting epigenetic reprogramming in PGCs has different implications than in pre-implantation embryo. Altered DNA methylation patterns produced due to exposure to environmental insults during the germ line epigenetic reprogramming can be transgenerationally-perpetuated (Koerner et al., 2009). This is because the germ line has the ability to transmit epigenetic marks to future generations (Skinner et al., 2010), a phenomenon termed “transgenerational epigenetic inheritance” (Daxinger and Whitelaw, 2012; Grossniklaus et al., 2013).

Recent investigations suggest that several diseases of common occurrence in human populations are not inherited in a Mendelian fashion, but through transgenerational epigenetic inheritance (Guerrero-Bosagna and Jensen, 2015). These environmentally induced diseases include obesity, polycystic-ovary syndrome or male fertility impairments (Anway et al., 2005; Skinner et al., 2013; Guerrero-Bosagna and Skinner, 2014). In all these examples, a maternal exposure occurred in which the germ line of the developing embryos was affected. When maternal exposure occurs, a true transgenerational effect is observed only in the F3 generation after the F0 maternal exposure, since it is the first generation not directly exposed (Skinner, 2008). Recent reports have shown that not only developmental exposures, but also juvenile or adult experiences could produce alterations in the germ line with consequences for future generations (Roth et al., 2009a). Examples include the exposure of juvenile male mice to low protein diets, producing alterations in the liver transcriptome of their offspring (Carone et al., 2010); fear conditioning of adult male mice with an odorant, which has consequences in the neural anatomy in the next two generations (Dias and Ressler, 2014); paternal stress in male mice, affecting the hypothalamic–pituitary–adrenal (HPA) axis and micro RNA expression in the offspring (Rodgers et al., 2013). Interestingly, in all these cases, the transgenerational transmission of these effects is mediated by paternal adult exposures followed by epigenetic alterations in the germ line. Studies in mammals have demonstrated that epigenetic changes triggered by stress exposure can also be passed on to the offspring (Gapp et al., 2014). In is important to highlight that while transgenerational effects derived from exposure of pregnant mothers affect the embryo, adult paternal exposures can produce transgenerational effects by potentially disrupting epigenetic processes during spermatogenesis (Rodgers et al., 2013, 2015; Milekic et al., 2015; Siklenka et al., 2015).

As for the mechanisms involved, influences from prenatal events can affect fetal gonadal development leading to transgenerational epigenetic modifications (reviewed by Chan et al., 2015). For example, it has been shown that environmental stressors can affect the developing fetal ovary, which results in long-term consequences through ontogeny, including predisposition to illness (Ren et al., 2017). Therefore, it is suggested that mammalian inheritance is highly prone to generate lasting epigenetic imprints driven by parental, prenatal and early-life stress, including malnutrition, trauma and maltreatment, leading to transgenerational consequences on neural mechanisms of cognition and emotion.

Nowadays it is widely acknowledged in our own species, thanks to studies applying the framework of the classic model of heredity (evaluated using the twins method), that the offspring of traumatized people have an increased predisposition toward developing posttraumatic stress disorder (PTSD), as well as mood and anxiety maladies (True et al., 1993). Moreover, it has been also demonstrated that post-traumatic stress disorder (PTSD) can have lasting effect during ontogeny and could even be inherited (Yahyavi et al., 2015). PTSD is a psychopathological reaction to an extraordinarily intense environmental stressor. There is evidence that hypothalamic-pituitary-adrenal axis changes in PTSD patients may also be evident in their offspring. One of the ways of measuring PTSD is by assessing cortisol levels. Interestingly, the offspring of parents with PTSD exhibited lower cortisol levels (King et al., 2001). Therefore, the authors suggested that the effect and biological vulnerability to PTSD might be transmitted across generations through maternal epigenetic programming during pregnancy (Yahyavi et al., 2015). By measuring PTSD using cortisol, researchers (Yahyavi et al., 2014) have provided the most important non-genetic biological evidence of the transgenerational transmission of PTSD in humans. This study included three different causes of parental PTSD: The Holocaust, the World Trade Center collapse, and maternal childhood abuse (Yahyavi et al., 2014; Yehuda et al., 2014). Furthermore, trauma and abuse in early life have been associated to allele-specific changes in methylation patterns of genes moderating the induction of psychosis (Klengel et al., 2012; Klengel and Binder, 2015).

In humans it is difficult to study the inheritance of epigenetic alterations. However, it has been possible to follow stress-exposed fathers developing PTSD and the subsequent effect on at least two of their filial generation. The transgenerational study of the horrible environmental stress that induced PTSD in Holocaust victims, and its influences on following generations, allowed to demonstrate for the first time how a PTSD event can be inherited epigenetically in a transgenerational fashion in our own species. Yehuda et al. (2016) studied a stress-related gene that has been associated with PTSD and depression (FKBP5), and demonstrated that people who suffered during the Holocaust presented DNA methylation modifications in this gene. This epigenetic influence was observed not only in exposed parents, but also in their offspring. Both Holocaust survivors and their descendants showed epigenetic changes at the same site of FKBP5 intron 7, but in the opposite direction. Holocaust survivors presented higher methylation than control parents, while Holocaust survivors' offspring showed lower methylation than the control generation. Moreover, in other mammals, such as in rats, it has been found that the transgenerational influence of stress on the offspring varied based on sex. Zaidan et al. (2013) have demonstrated that chronic, mild pre-reproductive stress (PRS) in adolescent female rats influences behavior and corticotrophin releasing factor 1 (CRF1) expression in the brain of the first filial generation. Furthermore, it was later demonstrated that the F2 offspring decreased CRF1 mRNA expression at birth, and exhibited altered anxiogenic behavior in adulthood (Zaidan and Gaisler-Salomon, 2015). It was found that corticosterone (CORT) levels were high in PRS females, as well as in the females from their F1. However, this was not observed in their F1 male offspring. Meanwhile, in the F2 generation CORT levels in PRS offspring also varied depending on the sex. These findings point out that PRS influences in adolescent females lead to behavioral modifications that extend, at least, to the second-generation offspring, and indicate that there is a transgenerational effect on endocrine function. These results suggest that PRS in adolescent females affects behavior and the HPA axis function across three generations, which highlights the importance of examining transgenerational effects of stress in both male and female offspring.

Therefore, stress-related epigenetic marks could be found in people undergoing stress during their ontogeny, with long term consequences on their psychological functioning, and these changes can even be preserved in the following generations as an intergenerational traumatic response (Mulder et al., 2017). In this context, among these environmentally-induced emergent influences on cognitive development, socioeconomic disparities have also been documented (Noble et al., 2015). Children from poor socioeconomic backgrounds are more prone to mental illnesses, such as depression, than their peers from wealthier families, and are also more likely to present cognitive problems (Noble et al., 2015). These children from impoverished families presented increased DNA methylation at the serotonin transporter gene, which predicts higher threat-related amygdala reactivity. Importantly, the serotonin transporter gene drives a number of autonomic responses to stress (Feder et al., 2009). These findings reveal that lower socioeconomic status acts as a stressor altering brain function. This pathway may represent a novel biomarker for the intervention against and prevention of specific behaviors associated with high-risk individuals (Swatz et al., 2017). Similarly, regarding the effects of the educational environment, it has been demonstrated that children exposed to bullying during school years are more susceptible to develop PTSD later in life, along with other mental illnesses such as suicidal tendencies and self-injury (Dhingra et al., 2016). It has also been found that people who suffer from bullying in their educational environment may also be involved in bullying episodes at work later in life (Nielsen et al., 2017). Likewise, workplace bullying is associated with PTSD symptoms, augmented physical, mental and health problems, burnout, increased intentions to leave, and reduced job satisfaction and organizational commitment (Nielsen and Einarsen, 2012).

**Figure 1:**
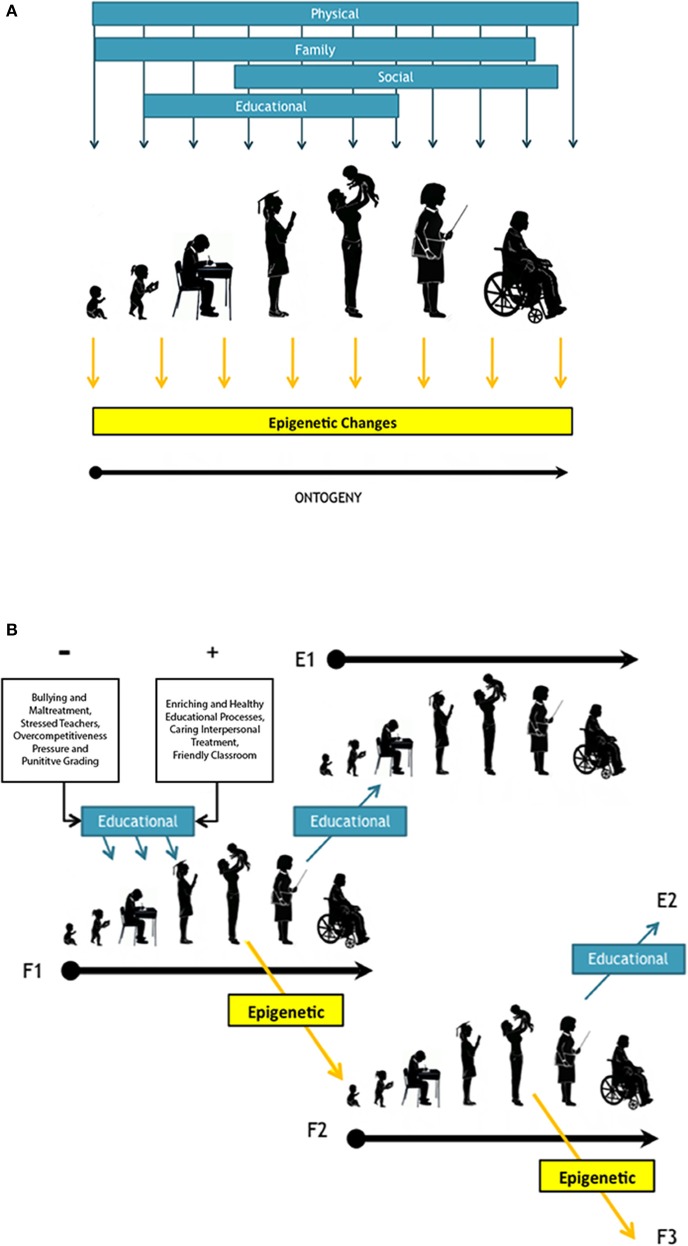
**(A)** Schematic representation of human ontogeny, from infant, toddler, child student, secondary student, adult with offspring, working adult (represented by a teacher), and elder adult. It is highlighted the environmental regulators influencing behavioral development through life in dark cyan arrows and rectangles. Among factors, it is considered Gottlieb's environmental regulator (1992) such as “physical” and “social” but “cultural” it is subdivided in to “educational” and “family” environment Together with this, we show in yellow arrows and rectangle the potential instances of epigenetic changes. **(B)** Transgenerational inheritance triggered by influences during ontogeny either due to environmental inputs or epigenetic changes. In cyan box and arrows it is highlighted the effects nourished either from negative (−) or positive (+) educational environment. “F1” represent the ontogeny of a first generation and “F2” and “F3” correspond to two consecutive filial generations. Meanwhile generation “E1” and “E2” represent non-filial ontogenies affected environmentally by educational influences (cyan boxes) provided by F1 and F2 respectively, such as teachers' impact over their students' ontogenies. Different educational influences, either − or +, are represented by a diagonal row reaching child development stages. Alternatively, it may be transferred to a following generations (F1 and F2) by transgenerational epigenetic inheritance (n yellow box and line).

Thus, violent interpersonal treatment and unhealthy educational processes, such as bullying, or mistreatment from a teacher, can be classified as a repeated traumatizing stimuli capable of leading to PTSD (Foa et al., 2009), with cultural as well potentially epigenetic inheritance effects. Therefore it is plausible to hypothesize that behind reiterated stress patterns there is a cascade of interactions across different of levels of biological organization that leads to changes in behavior, with consequences in adulthood (Nielsen et al., 2017) and the potential to be transgenerational perpetuated through epigenetic inheritance (Figure 1). Therefore, due to the potential long-lasting consequences of negative as well as positive stimuli on the ontogeny of our species, is paramount to protect our children from potential noxious influences during their development.

## The transient nature and reversibility of epigenetic changes

From a social point of view, and particularly in early education, it is very important to study the reversibility of epigenetic marks produced by negative environmental influences capable of leading to post-traumatic stress and other mental illness. There are multiple examples of reversibility in epigenetic marking in different model systems. For instance, in animals it has been demonstrated that DNA methylation normally occurs dynamically in relation to photoperiodic time (Stevenson and Prendergast, 2013). Similarly, in plants, it has been shown that DNA methylation epigenetic changes established by exposure to particular stress conditions can be gradually reverted and returned to epigenetic states similar to those of maternal plants once stress conditions are discontinued (Baránek et al., 2015). For instance, Dudley et al. (2011) have recently discovered in rats that offspring from high fat (MHF) nutrition mothers presented an altered regulation of liver development, a derangement that is detectable throughout early postnatal life. In these MHF-mothers' offspring, cell cycle activity showed significant G0/G1 arrest, linked with an hypomethylation of the hepatic cell cycle inhibitor Cdkn1a and an augmented expression of mRNA, correlated with a decrease both in liver weight and liver/brain weight ratio. The researchers proposed that these offspring are predisposed to long-term hepatic problems. However, recently Bertoldo and coauthors (Bertoldo et al., 2018) have described in rats that epigenetics changes could be ameliorated by exercise, stressing the reversibility of these changes.

Novel stressors may act as environmental influences on organisms driving the induction of epigenetic changes. It has been proposed that this begins with prompt, short-lived epigenetic changes that may ultimately lead to stable epigenetic marks in a multistep fashion. For instance, in mice early life stress (ELS) caused by maternal separation induced histone acetylations that altered the expression of the synaptic plasticity genes Arc and Egr1 in the hippocampus. These genes are involved in the development of limbic brain circuits. These fast epigenetic modifications are paralleled by raises in dendritic complexity and spine number of hippocampal CA3 pyramidal neurons in animals suffering ELS in early ontogeny. Therefore, ELS induces activation of synaptic plasticity genes, mediated by epigenetic mechanisms. These events represent early steps in the adaption of neuronal networks to a stressful environment; they program the developing organism and influence brain and behavior development (Xie et al., 2013).

In humans, it has been hypothesized that early in life, social environment may have a long-lasting effect on mental and physical health trajectories due to epigenetic marking of specific genes (Mehta et al., 2013; Hollar, 2016a,b). DNA methylation divergence can be linked to stable phenotypes (Szyf, 2011). This process may occur in post-mitotic tissues after birth, and in response to an external signal derived from the social environment. It has been suggested that suitable social and pharmacological interventions could upturn negative epigenetic markings originated by early life detrimental social stimulus (McGowan and Szyf, 2010).

Although there is no conclusive evidence demonstrating the reversibility of epigenetic changes caused by PSTD in humans, the brain has been shown to exhibit a plastic response in the aftermath of traumatic stress (Bremner et al., 2008; McEwen, 2008). Changes in environmental cues and some treatments involving antidepressant drugs have been shown to reverse the effects of stress on hippocampal neurogenesis, with PTSD subjects developing increased hippocampal volume after such stimuli (Bremner et al., 2008). Moreover, methylation signatures in genes related to responses to PSTD are currently being used for prognosis and the evaluation of symptoms in the treatment of patients with different mental conditions including PTSD, depression and anxiety (Mulder et al., 2017). These experimental approaches strongly suggest that psychotherapy may operate as an “environmental regulation” capable of amending an altered epigenetic state (Yehuda et al., 2013). In this context, it has been proposed that epigenetic marks may be potentially reversible. Therefore, epigenetic changes “may not only change for the worse, but with the right intervention, also for the better” as recently stated by Mulder et al. (2017).

Usually, in mammals, development programming is regulated by epigenetic modifications that are somehow permanent through the differentiation of cells and tissues. For example, in mice partaking in maternal nutrition, this phenomenon brings stable phenotypic consequences for the offspring (Morgan and Whitelaw, 2008). However, in the case of metastable epi-alleles (MEs), the epigenotype is stochastically established in the early embryo, which results in considerable inter-individual variation (Waterland et al., 2010).

Concerning human behavioral development, the existence of epigenotypic variation may also contribute to the understanding of the basis of the “differential susceptibility” theory, which proposes that youngsters vary in their susceptibility toward environmental regulators such as psychotherapy (Belsky et al., 2007; Ellis et al., 2011; Conradt et al., 2013). This may help to explain the variable success of psychotherapeutic interventions in children (Mulder et al., 2017). Considering current epigenetic research, it is possible to support the idea that epigenetically-induced modifications may regulate how an organism adjusts to changes in the environment, making it less reliant on structural DNA (Mulder et al., 2017). Inter-individual variation must be taken into consideration for the successful development of therapeutic interventions, thus, allowing children with different epigenetic susceptibility to overcome mental conditions. Moreover, the concept of “differential epigenetically-based susceptibility” should also be incorporated into the construction of benign educational strategies. However, in order to reach to this level of application of epigenetic knowledge, the role of epigenetic plasticity on learning, stress response and children's health must be further studied.

## Epigenome and epimutations affecting behavior

The relevance of epigenetic processes in synaptic plasticity, learning, and memory has recently been demonstrated in different model organisms (Mundinger, 1995; Kramer et al., 2011; Lester et al., 2011). Lately, it has been shown that memory stabilization in post-mitotic neurons is linked to epigenetic mechanisms during cell differentiation and development (Day and Sweatt, 2011a,b) where memory formation and maintenance correlate to changes in DNA methylation patterns (Day and Sweatt, 2011a). For instance, early life stress has been demonstrated to persist due to the influence of epigenetic processes on learning and memory capabilities (Day and Sweatt, 2011b; McClelland et al., 2011).

Recently, Isabelle Mansuy's group from Zurich University has demonstrated that traumatic stress can be inherited in mice. In an experiment in which newborn pups were separated from their mothers for a period of 2 weeks, the pups reacted in a very dramatic manner to the maternal separation, developing symptoms of depression and antisocial behavior when reaching adulthood (Gapp et al., 2014). Moreover, the offspring were incapable of facing adverse or novel circumstances. Interestingly, such traumatized mice preserved this altered behavior throughout life, and these aberrant behaviors were observed for the next three generations (Gapp et al., 2014). Also, evidence in humans has demonstrated that posttraumatic stress during infancy, besides modifying behavior and generating psychological disorders later in life may also be inherited to the descendants (Yahyavi et al., 2014).

Behavioral changes are also shown to be related to the disruption of genomic imprinting, which corresponds to epigenetic marks that are differentially established in each parental allele, inducing parental-specific expression in diploid cells (Reik and Walter, 2001). Due to imprinting, the expression of some genes is restricted to one of the alleles. The alteration of imprinting in these genes may contribute to the development of neurobiological disorders such as psychosis and autism (Isles et al., 2006; Úbeda and Gardner, 2010, 2011, 2012). Studies in rodents have described genomic imprinting related with parental care (Wilkins and Haig, 2003; Wolf and Hager, 2006). Regarding epigenetic trauma affecting behavior, it has been demonstrated that early-life adversity produces long-term epigenetic changes in the brain-derived neurotropic factor gene (BDNF; Roth et al., 2009b). In another study, unpredictable maternal separation has been shown to induce depressive-like behaviors and altered behavioral responses to adverse environments. Interestingly, these changes correlated to altered DNA methylation patterns (Franklin et al., 2010). Moreover, these behavioral alterations were also observed in the offspring of males affected by maternal separation. In spite of the fact that the offspring was reared normally, DNA methylation patterns linked to altered gene expression were also observed in these individuals (Franklin et al., 2010). Such evidence demonstrates the relevance of epigenetic mechanisms in relation to lifelong and potential transgenerational effects in gene expression and behavior, incited by early trauma and stress.

The aforementioned study by Gapp et al. (2014) also considered the number and type of specific ncRNA (microRNAs) expressed in the cells of adult mice exposed to traumatic conditions during early life in comparison to non-traumatized individuals (Gapp et al., 2014). Interestingly, traumatic stress demonstrated to alter relative amounts of microRNAs in blood, brain and spermatozoids. Some of these microRNAs were produced in excess while others were underrepresented in comparison with control animals. These changes resulted from deficient regulation of cell processes controlled by these microRNAs. As mentioned above, adult rodents behaved quite differently from controls following traumatic experiences during infancy, showing depressive behaviors. These behavioral symptoms were also observed in their offspring, despite the fact that these pups were never exposed to stress during their own ontogeny, suggesting that germ line epigenetic marks were alerted due to the paternal stress and such alteration was then inherited trough the spermatozoids (Gapp et al., 2014).

This stress-related microRNA imbalance in spermatozoids seems to represent a key finding in the mechanism behind transgenerational transmission of trauma, and generate new research questions such as “how does stress promote deregulation of small RNAs?” Most likely it is the end-point of a chain of events beginning with the production of stress hormone excesses (Gapp et al., 2014). Interestingly, in these experiments the metabolism of the descendants of stressed mice was also affected. For example, insulin and blood sugar levels were lower in these individuals than in the controls. This evidence represents the first demonstration that metabolic patterns affected by stress might also be transgenerationally transmitted through epigenetic inheritance.

Thus, it is becoming increasingly evident that the surrounding environment leaves epigenetic footprints on human brains, organs and also gametes. Importantly, when occurring in gametes, epigenetic changes may even pass to next generations (reviewed by Denhardt, 2017; Mulder et al., 2017).

These mechanisms and examples of transgenerational epigenetic inheritance on behavior and cognition underline the relevance of incorporating this knowledge not only as additional chapters in school teaching but in the development of educational policy. In this context, the consideration of the effects of environmental pressures on students, as well as the effect parents have on them, gain special relevance for the performance of pupils in formal education. The following section focuses on our ideas regarding this topic.

## Relevance of neurobiological epigenetics in education

Neuroscience and its application to education is an emerging interdisciplinary field that integrates brain functioning, pedagogy and education (Sigman et al., 2014). This convergence has triggered a true revolution in educational research and practice (Sigman et al., 2014). However, there is still a need to fill the gap between science and school life in order to incorporate these latest discoveries toward improving and effectively impacting formal education (Howard-Jones, 2014).

Long-term exposure, especially during childhood, to a stressful environment has been demonstrated to have pervasive effects on mental health, triggering epigenetic alterations related to several mental conditions (McGowan et al., 2010). Early life experiences have been highlighted as key environmental regulators of epigenetic processes in human biology including psychological and cognitive development (Hollar, 2016a). A recent review by David Denhardt (2017) points out that economic deprivation, drug abuse and stressful social interactions (such as social defeat and parental abuse, or maltreatment by a caregiver) have different impacts on cognitive processes and psychological stability, which are mediated by epigenetic processes (see also Roth et al., 2009a; Turecki and Meaney, 2016; Denhardt, 2017).

An interesting example of the potential effects of these environmental regulators in formal education can be explored looking at the formal education system in Chile, the most socio-economically unequal OECD country (Karim et al., 2016). During the last few decades, Chile has undergone a systematic privatization of education, based on a voucher-type system that has resulted in unfair disbursements and striking performance differences by students from different income groups (Parry, 1997). In Chile, students in public schools exhibit poorer educational performance than pupils from private schools (“Informe Nacional de Resultados” SIMCE 2012-2014), which correlates to a higher exposure to stressful factors for children in the public educational system (e.g., maltreatment, bullying, drug addiction; Tijmes, 2012; Maturana and Vargas, 2015). This is concordant with evidence showing that poverty is among the factors leading to PTSD and that these stress patterns have a negative effect on a child's cognitive abilities (Milan et al., 2013). This is of utmost relevance when considering the aforementioned studies highlighting the connection between epigenetic environmental regulators and behavioral development (e.g., Noble et al., 2015; Denhardt, 2017). Therefore, under the current status of inequity and educational segregation in the Chilean educational system, it is likely that students from impoverished families are being exposed to stress capable of producing related epigenetic alterations (potentially triggering PTSD) within the educational environment. Moreover, the environmental stressors may trigger altered epigenetic signals that would accumulate with long-term exposure (Figures 1A,B). These negative pressures, in turn, may result in the development of impairments on higher-order cognitive processes, as well as in mental health disorders (Swatz et al., 2017).

The affected abilities that have already been found in early-life stress epigenetic changes can be several: working memory, decision-making, self-control and goal-directed abilities, planning, judgment, impulse control and cognitive flexibility (Logue and Gould, 2014; Denhardt, 2017). The school-age environment can affect performance during adulthood. The existence of negative environmental regulators of cognitive development in schoolchildren may be linked to academic success and the development of antisocial behaviors. In a cross-sectional study of psychological disorders observed in young Chilean offenders, researchers found a prevalence of mental illnesses such as marijuana dependence disorder, major depressive disorder, and anxiety disorders. This investigation found also a connection between these mental disorders and childhood deprivation and maltreatment, and parental death and drug abuse (Gaete et al., 2017).

Bullying represents an important source of stress in school children (Idsoe et al., 2012). A study evaluating 16,410 secondary school students in Finland demonstrated that about one in ten schoolchildren reported being bullied on a weekly basis at school, representing a constant source of environmentally-borne stress in youngsters (Kaltiala-Heino et al., 1999). Consequently, It was shown that both the bullied and bullies themselves presented elevated levels of depression and suicidal ideation (Kaltiala-Heino et al., 1999). Similar patterns have been found in other countries such as the United States, where in a study covering 2,000 students from 30 different middle schools (6th through 8th grades), 20% of respondents reported seriously thinking about attempting suicide (19.7% of females; 20.9% of males), while 19% reported attempting suicide (17.9% of females; 20.2% of males) (Hinduja and Patchin, 2010). The frequent exposure of youngsters to a stressful, aggressive school environment (either as victims or bullies) is shown to negatively associate to syndromes such as depression, ideation, and suicide attempts (Brunstein et al., 2007). Also teachers working in public schools often present mental health related disorders such as depression, stress and neurosis (García, 2013). Despite the fact that the underlying epigenetic link was not considered in these studies, both in the case of depression and suicidal stress patterns, recent studies have detected epigenetic signatures in patients related to the environmental stress they have experienced (Farrell and O'Keane, 2016; Turecki and Meaney, 2016).

Because the action of epigenetic mechanisms is crucial during brain development and its functional configuration (Fagiolini et al., 2009), the consideration of the epigenetic component of brain dynamics during learning and memory formation is becoming of growing importance for educational neurosciences, both in research and application (Levenson and Sweatt, 2005; Gräff and Mansuy, 2008; Day and Sweatt, 2011a,b). On top of this ontogenetic developmental process are the epigenetic transgenerational effects discussed earlier, which means that the effects of environmentally-induced epigenetic alterations could be quite pervasive (Pembrey et al., 2014).

To paraphrase Susan Oyama, the configuration of the educational process it is a developmental system itself, where students and their surrounding environment constitute a unit of dynamic development (Oyama, 2000). This is why it is paramount to define what the objectives of formal education are, in order to start designing novel programs that take into consideration the neurobiological and epigenetic aspects, as well as students, their families, and their living conditions.

As mentioned above, many studies show the inheritance of environmentally induced nervous system pathologies (Johnstone and Baylin, 2010). However, Mendelian genetics has been unsuccessful in providing an adequate explanation for the inheritance mechanisms related to the etiology of these diseases. Nonetheless, knowledge gaps remain, since the molecular mechanisms involved in this transgenerational transmission are not fully understood (Mulder et al., 2017). The recent advancements in molecular technologies have represented a major step to disentangle the molecular mechanisms implicated in the inheritance of behavioral traits. We are now aware that epigenetic mechanisms play a preponderant role in this process, by connecting environmental stimuli to altered molecular function involved in disease etiology (Fenoglio et al., 2013).

In recent years mRNAs and micro RNAs have been found to play a major role in neural and synaptic plasticity (Burke and Barnes, 2006; Vo et al., 2010). The regulatory effects of micro RNAs over dendrite morphogenesis are examples of their action during early development (Kosik, 2006; Smalheiser and Lugli, 2009; Bredy et al., 2011; Fenoglio et al., 2013). Some small RNAs, such as siRNAs, show drastic increases during the early stages of learning, which have been suggested as relevant for the etiology of neuro-psychiatric diseases (Roth et al., 2009a; Smalheiser and Lugli, 2009; Spadaro and Bredy, 2012) Likewise, some long ncRNAs are involved in the transcriptional regulation of genes associated with neurological disorders such as Huntington's disease, schizophrenia, epilepsy, Prader–Willi syndrome, and Alzheimer's and Parkinson's Diseases (Vučićević et al., 2014). Such evidence demonstrates the relevance of the environment where early development takes place, and of transgenerational epigenetic processes, for the achievement of cognitive goals in formal education and learning (Galván, 2010). These findings also highlight the importance of considering the educational process as a developmental system (sensu Oyama), where parental health is a fundamental part of the student's inherited background. Certainly, early developmental experiences will establish the foundation where the bricks of further experience acquisition will be cemented. This developmental system, however, must consider both learning and its related cognitive process within an epigenetic dimension, allowing pupils to achieve educational goals while constructing their own set of meaningful topics within their brains. This can only be achieved in a suitable environment for brain development, especially in early stages of their structural organization.

Because epigenetic-related advances are having a significant impact on behavioral sciences and neurobiology, it is paramount that these emerging concepts are incorporated into philosophical and methodological aspects of teaching at the levels of primary and high school.

## Epigenetics within the classroom

Children are mainly subject to three types of environment during their developmental years: (1) the families, (2) their neighborhoods and (3) their schools. In these three types of environment, children can be subjected to positive and negative stimuli that will be very important in their learning process and personal formation. From kindergarten teaching up until leaving high school, children spend a great deal of time in classrooms, more so than in their houses and neighborhoods. For example, it has been widely documented that, early on in a human's ontogeny, the activity of hormones liberated as a response to stress, particularly glucocorticoids, can be modulated by social environmental conditions, such as sensible and interactive parental caregiving. For example, it has been demonstrated that social and educational influences by the end of first year of life can make newborns more resilient to several stressors (Gunnar and Barr, 1998; Gunnar and Donzella, 2002).

The experiences that students have within a school context are among the most critical predictors for their wellbeing and educational success (Wang and Eccles, 2013). It is at the school where they learn skills and project their future role in society (Hannah, 2013); it is also at the school where, by means of learning, the brain develops rapidly, establishing new neural connections. During these early stages of brain development, the role of the environment becomes of great importance for learning (Lenroot and Giedd, 2006; Saxe et al., 2009). It is during these critical stages where epigenetics may play an important role in education (Roth and Sweatt, 2011). Through negative environmental stimuli, some stress-related genes can be rapidly expressed and contribute to learning blockages. Teachers play a central role in establishing a positive and responsive classroom environment for children, which is important for promoting the development of social, emotional, and academic skills in students (O'Connor and McCartney, 2007). Interestingly, stress experienced by teachers can also be transmitted to their students (Spilt et al., 2011). It has been demonstrated that the occupational stress observed in teachers is linked to the physiological stress regulation of students (Oberle and Schonert-Reichl, 2016).

Studies suggest that epigenetic mechanisms are linked to learning and memory. For instance, DNA methylation and histone post-translational changes (methylation and acetylation) are capable of editing gene function or influencing interactions between the genome and core histones. These epigenetic modifications lead to chromatin remodeling, which ultimately defines transcriptional patterns in a variety of genes involved in memory formation and learning (Kim and Kaang, 2017). Moreover, the connection between the malfunction of epigenetic regulation and the occurrence of several mental disorders has become rather clear thanks to recent research (Rudenko and Tsai, 2014). For example, it is now known that many long ncRNAs are associated with genetic and developmental disorders related to learning disabilities and cognitive impairments (Vučićević et al., 2014). Moreover, the regulation of gene transcription mediated by microRNA has recently been found to be related to learning and memory in mice. In the same manner, miRNAs have been linked to the initial formation of fear memories in Dicer knockout mice (Dias et al., 2015).

Some educators are starting to pay attention to the importance of epigenetic mechanisms within the classroom. According to McEwen (2008) it is important for school staff and school policy-makers to grasp the key concepts of epigenetics, as it will probably soon be part of the scientific literacy in the classroom. This teaching practice has occasionally been used, for example when applying an active-learning approach to teaching epigenetic concepts and their consequences on heredity (Colón-Berlingeri, 2010). We hope that the consideration of these mechanisms by teachers in schools will also permeate to their educational practices in order to reduce stressful methodologies and improve educational health.

The teaching of epigenetic concepts and their possible consequences in inheritance has an academic value not only in the short term but also in the long term. We hope that a societal knowledge of epigenetics and its consequences in inheritance can be culturally transmitted across generations. This will generate the theoretical ground for initiatives aiming at reducing unfavorable environments that cause mental disorders such as PTSD. In addition, knowledge of the importance of epigenetics in government authorities will allow for the implementation of public policies to increase friendly environments not only in the classroom but also in society. Quoting Gabriela Mistral (1889–1957), Nobel Prize in Literature in 1945: “The future of children is always today, tomorrow will be late.”

## Discussion and future developments

The consideration of epigenetics provides neurosciences with new mechanisms for the understanding of the transgenerational inheritance of behavior (Gottlieb, 1992; Oyama et al., 2003), specifically regarding the acknowledgment of the connection between stressful situations and associated changes in gene transcription. Thus, the incorporation of an epigenetic framework gives novel dimensions and tools to neurobiological and psychological research to decipher the relevance of interactions between the different bidirectional coactional influences on organism' development: from environmental signals, behavioral plasticity, neural and endocrine phenotypic changes, chromatin marks and RNA modification, genomic imprinting and the genetic composition (reviewed by Meaney and Ferguson-Smith, 2010).

In order to appropriately understand the relevance of epigenetic processes in the classroom, it is necessary to consider research questions about the epigenetic components involved in the regulatory processes of our central nervous system (Day and Sweatt, 2011a). When education researchers achieve this, its transposition to educational policies cannot wait.

It is necessary to extrapolate these brain-developing factors to educational concepts used at all levels of education, from pre-school to Universities. Accordingly, actions must be taken within the classroom in order to ensure fair conditions for memory and learning development from early childhood to adulthood. We believe it should be a must to promote and foster adequate environmental conditions in educational institutions, backed by appropriate regulations, in order to ensure an enriching and healthy educational process. In this way all actors involved in the educational system will contribute to educational goals and to the prevention of cognitive and psychiatric disorders in students. This becomes even more important when considering the potential hereditary consequences of stress.

Statements such as “la letra con sangre entra” (literal translation: “bloody written characters are better learnt”) which is the Spanish equivalent to “spare the rod spoil the child” “are classically repeated by the South-American Sarmiento's school of education (reviewed by Carli, 2001; Escudero, 2014). Such philosophy is still alive, since “rigor and stress” are generally seeing as a positive factor in contributing toward the learning process and behavioral development of youngsters. The consequences of a punitive educational regime may be even more acute in poorer segments of the population, at is the case in Chile, where orphans and young offenders are often sent to homes belonging to the National Service for Minors (Servicio Nacional de Menores, SENAME). Such centers were recently found to be a nationwide hotbed for child abuse due to caregiver maltreatment (Human Rights Watch World Report, 2017). As we have pointed out, insult and stressors derived by neglect and abuse at early life may lead to long-term consequences in youngsters (Gunnar and Donzella, 2002). The extremely stressful environment experienced by young offenders may be related to high incidence of mental disorders, as recently reported by Gaete et al. (2017). However, no investigation has addressed the link between epigenetic mechanisms and psychological consequences for these victimized children.

Based on the negative effects of traumatic experiences for the cognitive process and mental health of students (Prinzie et al., 2004), and on the hereditary consequences suggested by the findings reviewed here, we believe aggressive and stressful educational environments are even more pernicious than was previously thought, before the recent boom in epigenetic research. As such, special care should be taken in the development of future teachers and school educators in this respect. Serious efforts must be taken in order to raise awareness of the different influences shaping the outcomes of school teaching. Furthermore, it will be imperative in the near future to deepen the understanding of the influences of these experiences on cognitive development during educational processes, and their potential to generate transgenerational effects. We believe that the “Epigenetics of Education” has to be considered as part of the equation in formal education.

Inadequate learning environments may affect epigenetic marks involved in developmental processes, leading to brain changes. Based on current evidence from epigenetic studies, these stress factors and unhealthy environments may alter the formation of neural circuits, affecting the acquisition of abilities linked to learning. Moreover, the possibility exists that gametic epigenetic marks are altered in students as consequence of environmental stress, and therefore that the effects of such stressful conditions would be transgenerationally transmitted, with potentially disastrous consequences for the mental health of future generations. Epigenetic processes are a scientific fact and must be incorporated into our educational reality in order for us to be able to consider their potential value in favor of a better educational system. Recent progress has to be acknowledged, such as The Moore Institute School of Medicine with its “Let's Get Healthy” initiative (www.letsgethealthy.org), which incorporates the epigenetic dimension into its middle school program. We propose that efforts must be concentrated on developing multidisciplinary research and a dialog between educators and scientists toward building an applied field focused on education that accounts for environmental influences, as well as for developmental and epigenetic processes: i.e., Educational Epigenetics.

## Final epistemological considerations

Current evidence provided by the study of epigenetic influences on behavioral development and transgenerational consequences through cultural as well as epigenetic inheritance highlights the dynamic nature of organisms' plasticity. As stated by Chan and coauthors: “It is this inherent developmental plasticity of an organism that allows it to respond to cues that will ultimately determine the adult” (Chan et al., 2015). Contemporary thinkers of the construction of cognition remark the dynamic essence of behavioral processes including learning (Oyama et al., 2003). Following the thread lead by Waddington and Piaget (Waddington, 1953; Piaget, 1969, 1970), Oyama also refused to make the distinction between nature and nurture in the development of behavior, and to give a preponderant role to allelic genetic mechanisms to explain human psychology. Instead, she defined cognitive processes as developmental systems, stressing the existence of dynamic emergent controls that help to understand the acquisition of phenotypic modifications within both the evolutionary and ecological contexts (Oyama, 2000). These controls emerge from interactions at different hierarchical levels during cognitive and behavioral developments (*sensu* Gottlieb, 1992). From our perspective, learning and formal education fit Oyama's notion of developmental systems and are, therefore, subjected to this dialectical flux between hierarchical levels. These active influences may not only define behavioral dysfunctions and stressed phenotypes, but may also have phenotypic and hereditary consequences for our own species (Frías-Lasserre and Villagra, 2017). Such regulatory dynamics have been demonstrated to exert paramount impact on maternal and child development from nutrition to behavioral and social interactions (Hollar, 2016a,b).

Therefore, epigenetic effects over an individual ontogeny may firstly influence its physiology and structural development and, through its ontogeny (Figure 1A), these changes may accumulate environmentally induced epigenetic modifications that ultimately may pass across generations and individuals' lifespan (Figure 1B; Hollar, 2016a). This has been clearly evidenced in patients suffering from PTSD resulting from different environmental insults such as war, child abuse, and famine survivors (Lumey et al., 2007; Yehuda and Bierer, 2009). More “subtle” but repeated stress factors however, have been also been considered to be environmental regulators of epigenetic influences. Within this context, educational environment and socioeconomic factors must also be considered as extremely relevant for behavioral development and learning (Hackman et al., 2010). All these considerations, based on current experimental evidence, should not only fuel further inquiry and basic research, but should permeate social policies, as well as political decisions, in order to protect the wellbeing and future of the cognitive development of our own species.

## Author contributions

DF-L main hypothesis and writing developed. CV writing, bibliographic review and figures. CG-B writing, reading and suggestions in the final version.

### Conflict of interest statement

The authors declare that the research was conducted in the absence of any commercial or financial relationships that could be construed as a potential conflict of interest.
